# A Rare Case of Intramediastinal Humeral Head Fracture Dislocation Through the Thoracic Inlet

**DOI:** 10.1002/ccr3.70420

**Published:** 2025-04-14

**Authors:** Nicola Rotolo, Cecilia Colombo, Marco Puricelli, Fabio Berizzi, Luca Filipponi, Andrea Pautasso, Fabio D'Angelo

**Affiliations:** ^1^ Thoracic Surgery Unit, Department of Medicine and Technological Innovation, Research Center of Minimally Invasive Surgery University of Insubria Varese Italy; ^2^ Orthopaedic and Traumatology Department, Ospedale di Circolo e Fondazione Macchi University Hospital Varese Italy; ^3^ Division of Orthopaedics and Traumatology, Department of Biotechnologies and Life Sciences University of Insubria Varese Italy

**Keywords:** chylothorax, humeral head fracture dislocation, reverse shoulder replacement, thoracic trauma, video thoracoscopy

## Abstract

Intramediastinal fracture dislocation of the humeral head due to a blunt trauma is very rare. It may be accompanied by local and systemic injuries associated with high‐energy trauma. Only one case has been reported in the literature; therefore, an appropriate treatment modality remains unclear. We present a case of a 71‐year‐old woman involved in an accidental fall who sustained an intramediastinal humeral head fracture dislocation through the thoracic inlet, without damaging the chest wall, causing the thoracic duct rupture and requiring three consecutive surgical procedures as treatment.

**Summary**

- Although a rare event, the intramediastinal displacement of the humeral head should always be suspected in major shoulder traumas due to the severe and potentially fatal consequences it may have.
- A chest CT scan is mandatory for diagnosis.
- A multidisciplinary approach is essential for its management and treatment.

## 1 Introduction

Intrathoracic and intramediastinal fracture dislocation of the head of the humerus (HH) is rare, with 28 cases reported in the literature. Due to its potential and significant risk of devastating complications, such as vascular, nervous, and pleuro‐pulmonary lesions (fatal hemorrhage due to subclavian and brachiocephalic arteries or veins lesions; thoracic duct lesion; lesions of cervical and brachial plexus, and phrenic and vagus nerves; emo‐pneumothorax), it is crucial to remove it from the “host” anatomical site. First described by West in 1949 [1], it has been reported in the literature only in 28 cases [1, 2, 3, 4, 5, 6, 7, 8, 9, 10, 11, 12, 13, 14, 15, 16, 17, 18, 19, 20, 21, 22, 23, 24, 25, 26, 27, 28] as well as a retroperitoneal [29] and a contralateral hemithorax dislocation [11]. Wiesler and coworkers reported a similar case of HH displacement into the thoracic inlet, stopping its movement over the first rib, neighboring the pleural cupola, without chest wall injury [30]. To date, only one intramediastinal dislocation of the HH is reported by Agarwalla; the authors described a case of an intramediastinal fragment of the HH placing into the aortopulmonary window through the first and second ribs with injury of the thoracic wall [31]. Here, we have documented a remarkable case involving an HH fracture dislocation into the mediastinum, without internal chest wall damage, after a household left shoulder trauma, as the first case reported in the literature, to describe its treatment and complications management. Our case has been reported in line with the CARE criteria [32].

## 2 Case History and Examination

A 71‐year‐old woman was admitted to the emergency room of our hospital with left shoulder chest pain, following an accidental fall at home, slipping while doing household chores. She denied loss of consciousness or injury to other extremities. Physical examination revealed the awake patient in no acute distress, breathing comfortably, and hemodynamically stable. On thorax auscultation, there were decreased breath sounds over the lower chest and dullness in the same area, bilaterally. At the upper left limb's evaluation, functional impotence of the left shoulder with no deficit to the peripheral nerves, a locoregional large ecchymosis, and severe pain on palpation of the shoulder and neck were found.

## 3 Methods

### 3.1 Diagnosis and Investigations

**FIGURE 1:**
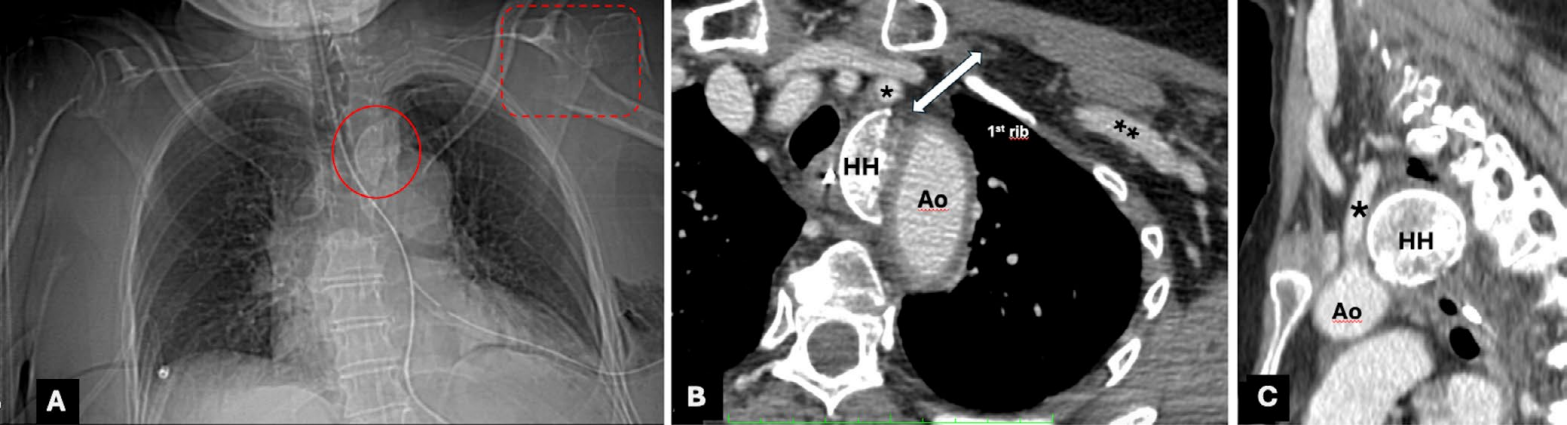
Chest x‐ray (A): Intramediastinal dislocation of humeral head (red circle) after its post‐traumatic fracture (box red dotted). Axial CT chest scan (B): Intramediastinal position of the humeral head, medially to the aortic arch (Ao), posteriorly to the left subclavian artery (asterisk), and laterally to the trachea. The bidirectional white arrow indicates the entry route into the mediastinum. Subclavian artery (double asterisk). Coronal CT chest scan (C): The bone fragment (HH) in the angle formed by the aortic arch (Ao) and the origin of the left subclavian artery (asterisk).

The chest X‐ray and computed tomography (CT) chest scan showed fracture and displacement of the left proximal humerus (four parts of Neer's classification) and a compound fracture of the glenoid with displacement of the HH in the upper mediastinum, between the upper margin of the aortic arch and subclavian artery, laterally to the trachea and anteriorly to the esophagus (Figure 1), with simultaneous fracture of the first left rib, with multiple bone fragments adjacent to the glenoid and in the space between the clavicle and the first left rib. No radiological signs of bleeding were revealed at the CT chest scan. Also, bilateral pleural effusion was identified. The CT chest scan did not reveal any other pathological conditions in the examined area.

## 4 Treatments

### 4.1 First Treatment

**FIGURE 2:**
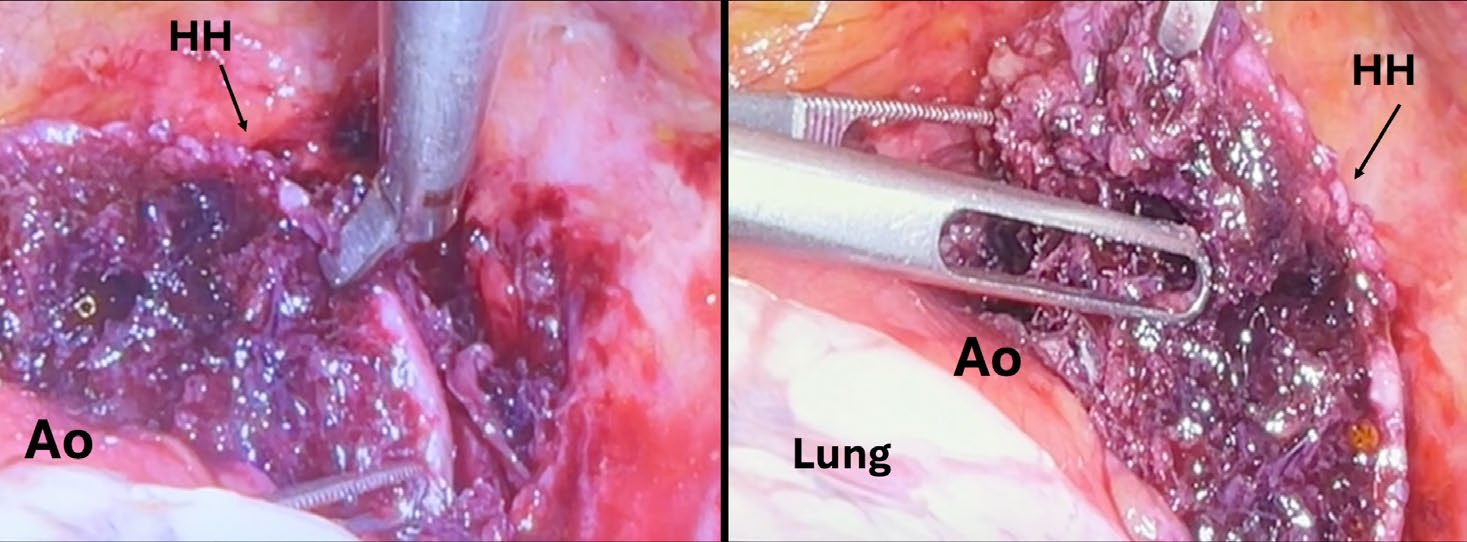
Intraoperative view of a fragment of the humerus head (HH) close to the aortic arch (Ao), gently remotion with two graspers.

**FIGURE 3:**
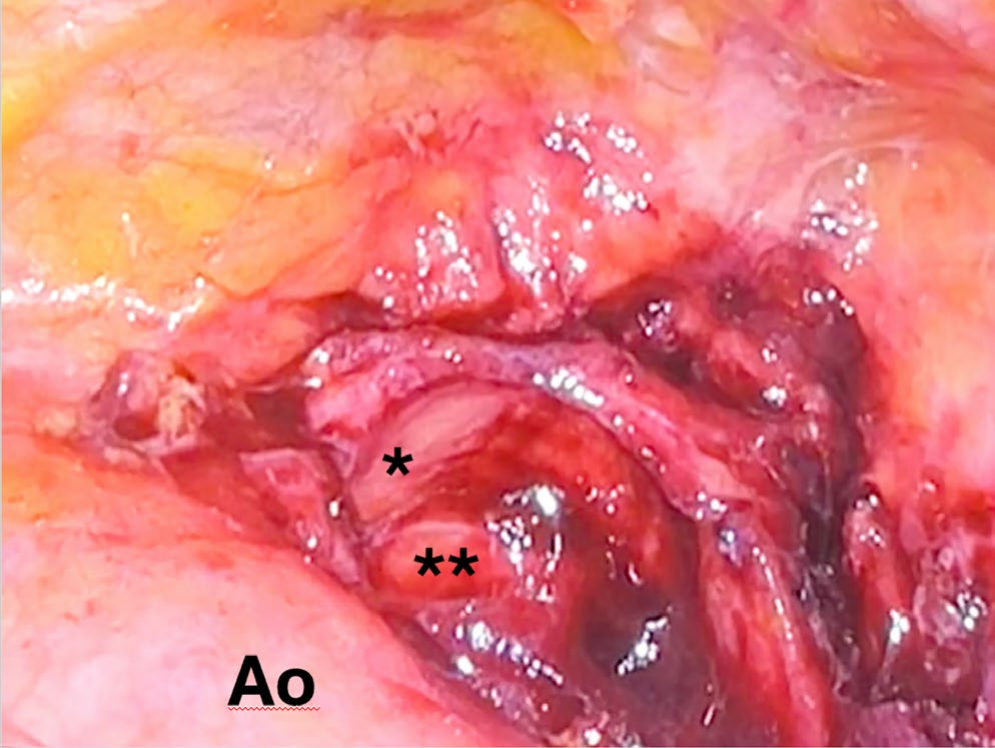
Surgical site after fragment removal. Ao, aortic arch. *Left subclavian artery. **Left carotid artery.

**FIGURE 4:**
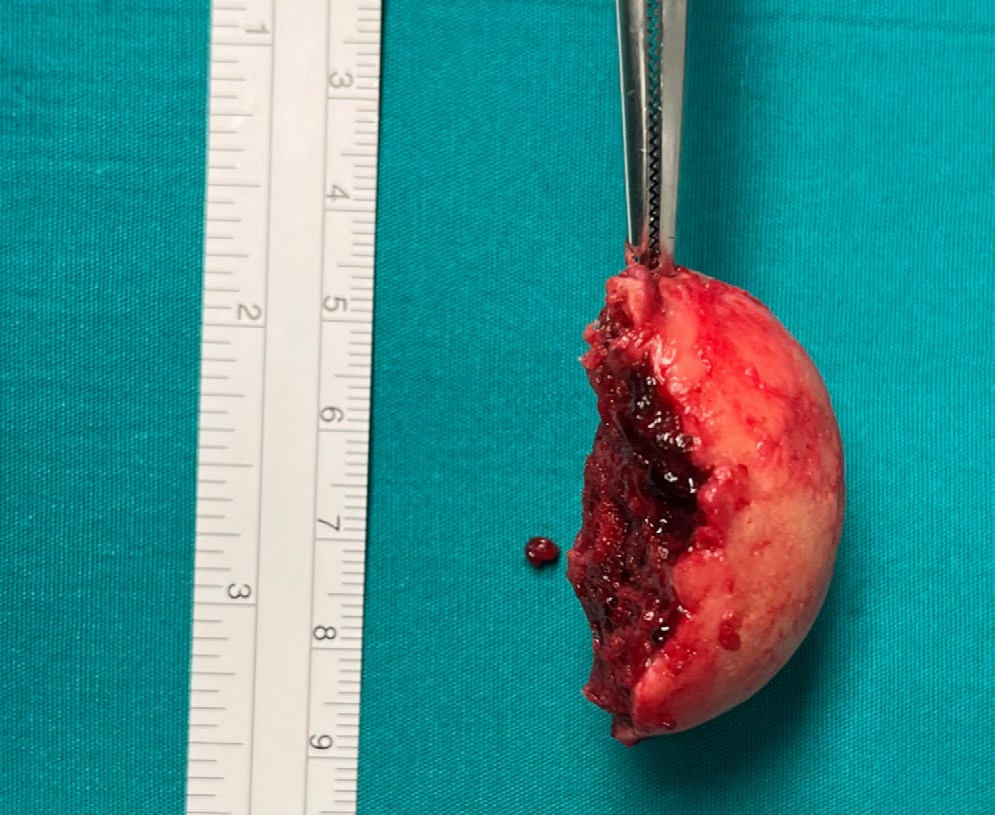
Fragment of a humerus head of 4 cm in diameter.

The placement of a bilateral pleural drainage allowed the evacuation of 1000 mL of chylous fluid from the right hemithorax and serum from the left hemothorax. After a multidisciplinary evaluation (thoracic surgeon, orthopedic, anesthesiologist, and resuscitator), the patient was referred to surgery to remove the fragment of mediastinal HH as the first step 4 days after the trauma and afterward to orthopedic surgery. The patient was placed in the semi‐supine position up to 30° with a roll placed under the ipsilateral chest. A left triportal video‐assisted thoracoscopic surgery (VATS) was performed via two 5‐mm ports (through the third intercostal space in the anterior axillary line and the sixth intercostal space along the midclavicular line) and one 10‐mm port (sixth intercostal space in the mid‐axillary line, camera port) with CO_2_ insufflation using a pressure limit of 8 mmHg (surgical access for the thymus gland). During the inspection of the pleural cavity, we surprisingly checked the integrity of the entire internal thoracic wall and its parietal pleura, demonstrating the doorway of the bone fragment into the mediastinum through the thoracic inlet. After opening the mediastinal pleura, we identified the fragment of the humerus located in the anterior mediastinum, on the upper edge of the aorta. We used two graspers to gently move the fragment (Figure 2) before its slow extraction since the rough face of the HH was turned toward the aortic wall to avoid vascular (aortic arch and subclavian artery) or tracheal lesions, the structures it was connected to (Figure 3). The fragment was removed without complications although it was necessary to widen one of the video‐thoracoscopic ports to extract it from the chest (Figure 4). The likely avascular necrosis of the fragment rendered it unsuitable for replanting. A 16 French chest drainage was placed in the pleural cavity to re‐expand the collapsed lung. The postoperative course was uneventful, and subsequently, 4 days after thoracic surgery, according to anesthesiologists, it was decided to carry out the orthopedic surgery planning for a reverse total shoulder arthroplasty. The reasons why orthopedic surgery was not planned on the same day as thoracic surgery and why a staged treatment was chosen instead are as follows. First, the complexity and duration of procedures; combining them into a single operation could result in a long surgical duration, increasing the risks of complications such as bleeding, infection, and thromboembolic events. Second, thoracic surgery can impact respiratory and cardiovascular functions, which may compromise the patient's ability to tolerate a second major procedure like orthopedic surgery. Staging the surgeries allows the patient to recover partially from the first procedure, reducing the overall physiological burden.

### 4.2 Second Treatment

**FIGURE 5:**
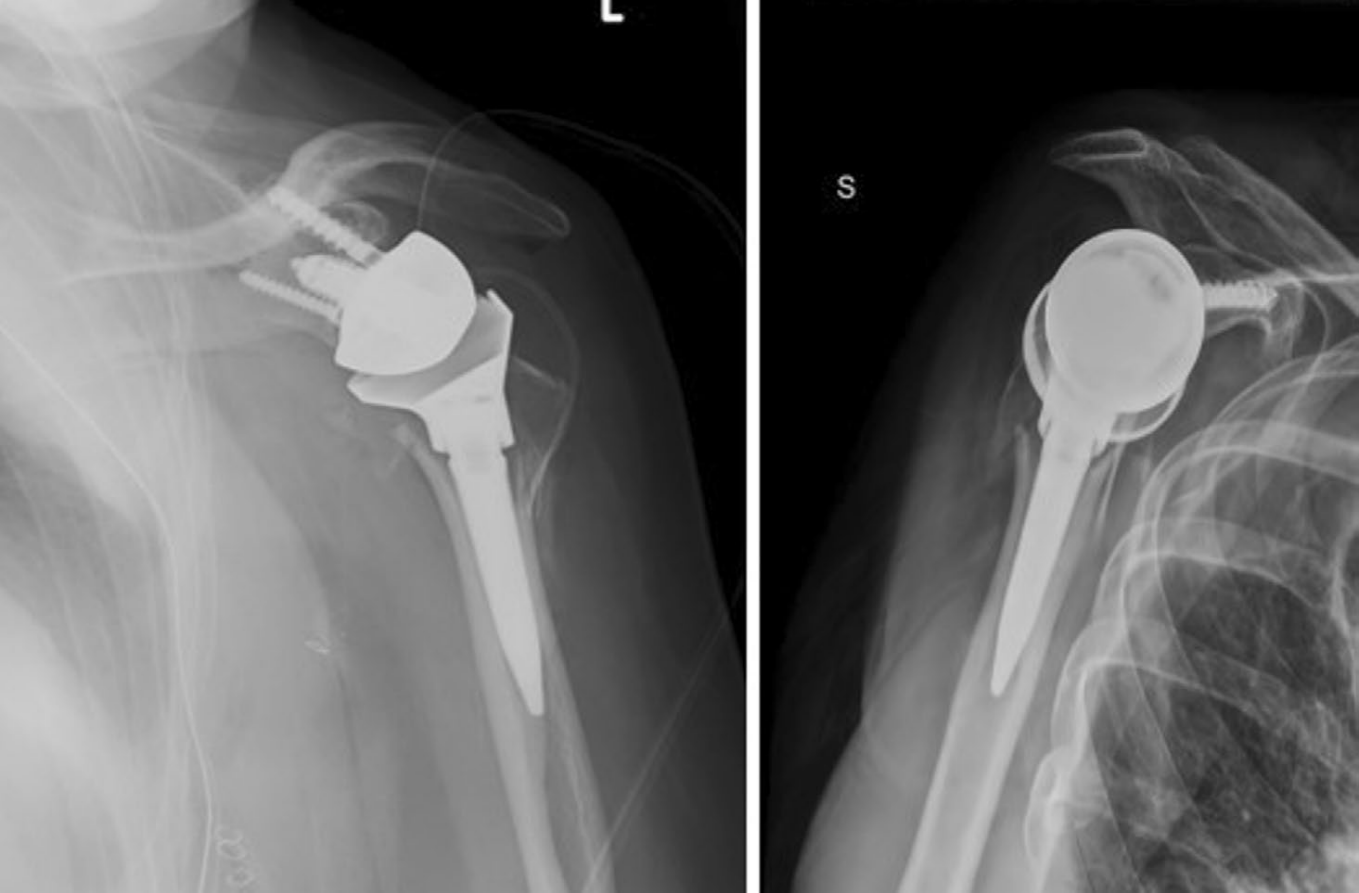
Postoperative left shoulder antero‐posterior and y‐view x‐ray: Lima SMR reverse shoulder replacement.

The surgery was performed under general anesthesia and interscalene brachial plexus block with the patient in a beach chair position. A deltopectoral approach was performed. After tenotomy of the long head of the biceps, the lesser and greater tuberosities were secured with stay sutures on the subscapularis and supraspinatus tendon. A complete soft tissue release around the glenoid was performed. After preparation of the glenoid surface, a metal back was implanted (LIMA Small‐R fixed with two screws #6.5 × 30 mm and #4.5 × 24 mm) with a 36 mm glenosphere (Lima Corporate S.p.A., Udine, Italy). The humeral canal was reamed, a #11 humeral stem was implanted, and a liner was inserted. After the range of movement (ROM) evaluation and stability testing of the implant, tuberosities were reduced and secured to the prosthesis, to the diaphysis, and to each other with “interlocking sutures” (Figure 5). A drainage was inserted, and after skin suture, the arm was placed in a sling. The postoperative protocol consisted of passive exercise with ROM limited to elevation and abduction of 90° for the first 4 weeks, and then progressive passive and active exercises without ROM limitation. Following the introduction of food, on the third postoperative day, she developed bilateral loculated pleural effusion detected by a chest CT scan, which required placement of bilateral pleural drainage with high‐volume chylous leakage daily, despite being treated with fasting and total parenteral nutrition.

### 4.3 Third Treatment

Because of the failure of conservative management and the worsening clinical status, we performed a bilateral uniportal VATS, right side first, to ligate the thoracic duct successfully. The surgical procedures have been preceded by an infusion of indocyanine green into an inguinal lymph node (0,5 mg per kg) to easily identify the thoracic duct intraoperatively, closed with interrupted sutures placed as low as possible in the chest, close to the diaphragmatic hiatus. Afterward, the patient underwent left uniportal VATS to toilet the chest cavity and to perform a pleurodesis with talc poudrage. Two 24 French bilateral chest drainages were inserted.

## 5 Conclusion and Results

The postoperative course was uneventful, with a prompt healing of clinical conditions. She was discharged on postoperative day 21 and referred for formal rehabilitation therapy 3 weeks postoperatively. A staged rehabilitation approach was scheduled and carried out in a specialized center to ensure proper healing, mobility restoration, and strength recovery. The rehabilitation program was conducted in several phases: passive ROM (where the therapist moved the arm) to maintain mobility without straining the shoulder, gentle active‐assisted movements (the patient began using their unaffected arm or a device to assist with gentle active ROM exercises), shoulder strengthening (isometric) light exercises to avoid atrophy of shoulder muscles (contracting the muscles without moving the joint), and finally, gradually reintroducing light daily activities.

## 6 Follow‐Up

Two years after treatment, the patient is in good clinical condition. Her shoulder ROM consisted of 120° of flexion, 100° of abduction, 30° of external rotation, and internal rotation at the level of L1, and she has returned to being independent in her daily activities.

## 7 Discussion

**FIGURE 6:**
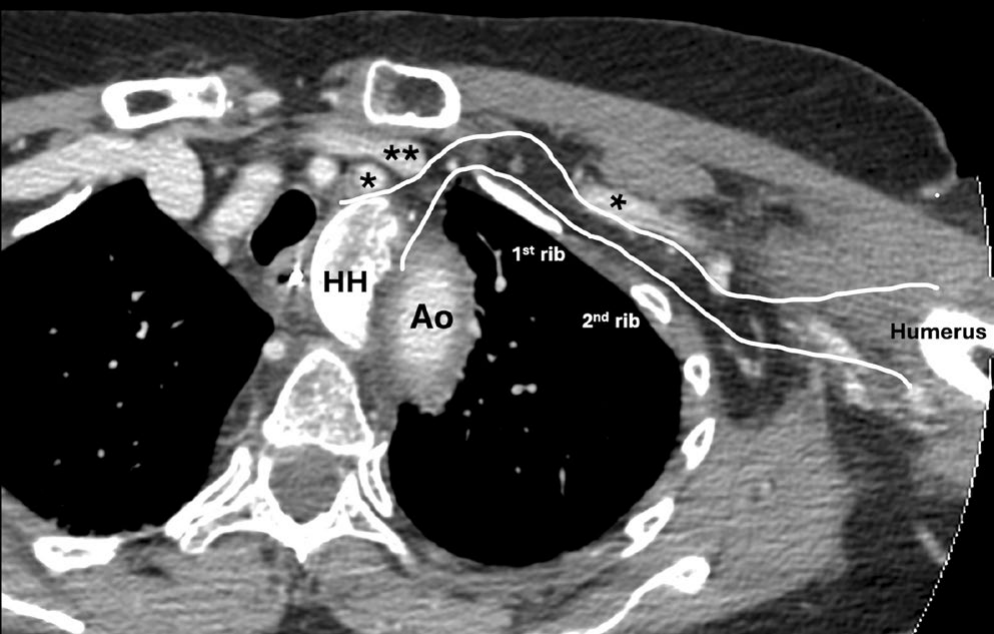
Route (white lines) followed by the bone fragment (HH) above the first rib. Ao, aortic arch. *Left subclavian artery. **Left brachiocephalic vein. None of these vascular structures have sustained injuries except for the thoracic duct.

**FIGURE 7:**
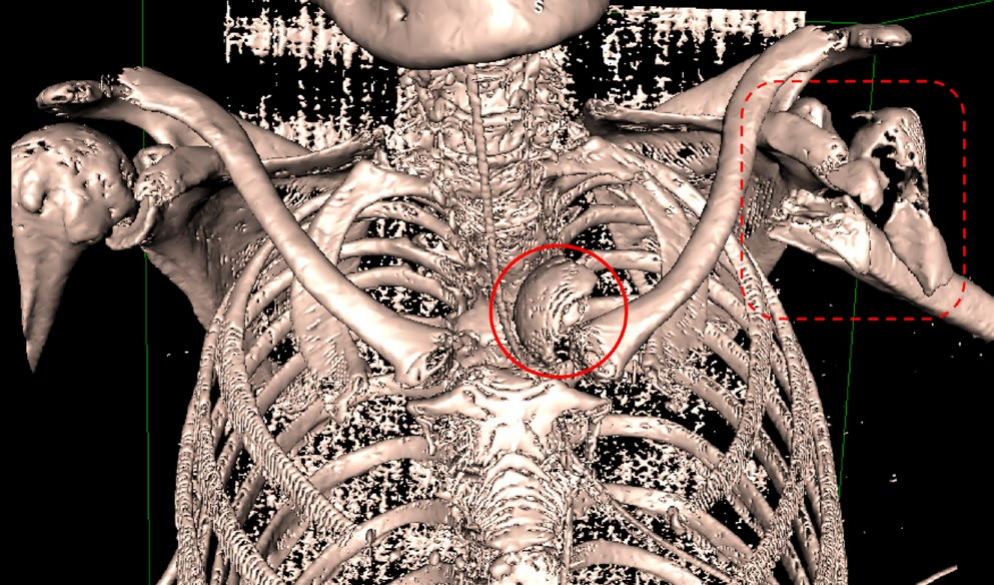
A suggestive three‐dimensional surface rendering image from a chest CT scan shows the dislocation of the HH into the mediastinum (red circle) and the HH fracture (dotted red box).

Our case is the first case of intramediastinal displacement of HH through the thoracic inlet, without injury to the chest wall, with rupture of the thoracic duct and right chylothorax, requiring a left video thoracoscopy to remove it and subsequently bilateral VATS to treat the chylothorax. The fragment reaches the mediastinum by passing below the left subclavian vein and artery, above the pleural dome, without opening the parietal pleura, and positioning itself close to the aortic arch, the left subclavian artery, the brachiocephalic vein, and the trachea, medially (Figure 6). A suggestive three‐dimensional surface rendering image shows the dislocation of the HH into the mediastinum (Figure 7).

The most common causes of injury were from a fall (simple fall, or downstairs or from a height) and road accidents, such as motor vehicle accident or a pedestrian being struck. Several theories have been proposed over the years to explain the mechanism of this kind of fracture, and all the authors agree that it is unlikely that a single traumatic force could cause this injury [8]. Probably, the mechanism of injury is a high‐impact trauma with the humerus in abduction and external rotation; a diastasis of the first and second ribs and the dislocation of the HH into the thoracic cavity are likely caused by a further force along the axis of the humerus. Subsequently, the abduction of the humerus results in a fracture of the surgical neck of the humerus, and the degree of displacement into the thoracic cavity and mediastinum depends on the magnitude of the traumatic force [31].

The clinical presentation may vary depending on the case: respiratory distress, rib fractures, ipsilateral ecchymosis, subcutaneous emphysema, hemothorax, or pneumothorax may be frequently found and should raise suspicion of humeral head displacement into the thoracic cavity [31]. The diagnosis is radiological and involves, in addition to the radiography, CT scan of the shoulder and thorax, which are the imaging of choice also to show the associated lesions (vascular and/or nervous) [28, 31]. As a rare injury, there is no consensus on treatment; several treatment methods were described in the literature, and the cases are treated on a case‐by‐case basis, individually, depending on several factors such as fracture pattern, patient age, preinjury activity level, soft tissue status, and comorbidities that play a major role. Some cases have been treated nonoperatively, without consequent complications due to the persistent intrathoracic location of the humeral head [3, 7, 8, 19, 21]. Many cases have been treated operatively instead, with the removal of the displaced humeral head and subsequent shoulder arthroplasty or hemiarthroplasty [1, 2, 10, 11, 12, 13, 14, 15]. In some cases, an open reduction–internal fixation (ORIF) of the fracture has been realized [4, 5, 6, 9, 16, 29], even if this modality of treatment may be associated with avascular necrosis [6]. The persistence of the humeral head in the thoracic cavity or mediastinum and its possible migration increases the risk of damage to surrounding structures, such as lung parenchyma, great vessels, nerves, and, as in our case, the thoracic duct. Our case is the first case of chylothorax due to thoracic duct rupture during migration of bone fragments. Hopefully, both the subclavian and jugular veins remained uninjured.

Remotion of the fragment is mandatory to avoid potential intrathoracic complications [13, 15, 31]. Consultation with cardiothoracic surgeons is necessary [31], and if the HH cannot be extracted through the injury tract, the surgery must be performed through a thoracotomy or thoracoscopic approach: the decision may be dependent on which structures are damaged [31]. In our opinion, VATS represents the best choice because it is a less invasive method and seems to guarantee a better exposure and control of the operating field. With intrathoracic or intramediastinic displacement, the humeral head is devitalized from its blood supply, which increases the risk of avascular necrosis [31], so shoulder arthroplasty is an efficient treatment modality for joint reconstruction compared to retention and fixation of the displaced fragment and provides the optimal outcome [15, 31].

To conclude, the intramediastinal displacement of the humeral head is a very rare event that can occur in high‐impact traumas occurring with the humerus in an abducted position, rotated externally. It should always be suspected in cases of shoulder trauma. The diagnosis is radiological by CT scan that shows any associated lesions (vascular and/or nervous). Surgical removal is mandatory, and the VATS seems a valuable and effective approach, both for better exposure and control of the operating field.

## Author Contributions

**Nicola Rotolo:** conceptualization, supervision, writing – original draft, writing – review and editing. **Cecilia Colombo:** writing – original draft. **Marco Puricelli:** data curation, writing – original draft. **Fabio Berizzi:** writing – review and editing. **Luca Filipponi:** data curation. **Andrea Pautasso:** data curation. **Fabio D'Angelo:** supervision.

## Ethics Statement

The authors have nothing to report.

## Consent

A written informed consent was obtained from the patient for the publication of this report and accompanying images.

## Conflicts of Interest

The authors declare no conflicts of interest.

## Data Availability

As a case report, data sharing is not applicable to this article as no datasets were generated or analyzed during the current study.
